# Brainstem evoked response in bus drivers with noise-induced hearing loss

**DOI:** 10.1016/S1808-8694(15)30529-2

**Published:** 2015-10-18

**Authors:** Adriana Silveira Santos, Ney de Castro

**Affiliations:** 1 MSc in Otorhinolaryngology – Medical Sciences School - Sta. Casa de S. Paulo; ENT physician - Hospital Sto. Antonio, Salvador, BA; 2 PhD in Medicine, Adjunct Professor

**Keywords:** acoustic trauma, evoked response audiometry, hearing loss, noise-induced, brain stem

## Abstract

Studies carried out by Brainstem Evoked Auditory Potentials (BEAP) in Noise-Induced Hearing Loss (NIHL) workers show different results in relation to neuronal involvement, not involving bus drivers as study object.

**Aim:**

to use BEAP in a prospective case/control clinical study to check whether or not there is neural auditory pathway involvement in bus drivers with NIHL.

**Materials and Methods:**

we selected 50 bus drivers between 27 and 40 years with mild to moderate NIHL, and 20 individuals between 29 and 40 years with normal hearing and without prior history of noise exposure. BEAP tests were carried out and the traces were analyzed.

**Results:**

in the NIHL group, the auditory thresholds in 3, 4 and 6 kHz were significantly higher in the left ear. In the NIHL group, potentials PI, PIII and/or PV were not present in a small number of the individuals; we observed a statistically significant increase in PI, PIII and PV absolute latencies, (LIP) LIP I-III interpeak latencies, bilaterally and LIP I-V in the left ear.

**Conclusion:**

in the NIHL group, besides sensorial injury, changes in BEAP latencies suggest an early functional injury of the first auditory pathway afferent neuron.

## INTRODUCTION

Noise is one of the most prevalent noxious physical agents present in the work environment today, and one of the major problems found in factories, means of transportation and in the very getting together of people.

Prolonged exposure to high intensity sounds produces successive changes to inner ear structures, initially transitional and later permanent. The final result is damage to the sensorial hair cells and its highest expression is the destruction of most of the organ of Corti, consequently causing noise-induced hearing loss (NIHL). Experimental studies showed the involvement of neural structures in NIHL^1^, ^2^, ^3^. Such studies have also shown, besides degenerative alterations caused to the outer and inner hair cells, the involvement of neuronal synapses near sensorial cells and the degeneration of afferent fibers of the cochlear nerve. These authors suggest two hypotheses which could explain the neuronal damage: neural hyperactivity by the excessive acoustic stimulation and/or neuronal response to the hair cells degeneration.

The NIHL is caused when the individual is exposed to noise intensity above 85 dB of Sound Pressure Level (SPL), eight hours per day, regularly, for a period of many years, usually setting in the first five years of exposure^4^. Its main characteristics are: sensorial, irreversible and, in most of the cases, bilateral and symmetrical. As to the spectrum of frequencies reached, NIHL happens predominantly in the frequencies of 3, 4 or 6 kHz and, as noise exposure continues, it extends to the adjacent frequencies, which take longer to be involved.

NIHL is seen and studied in different professional occupations – urban bus drives among them. There are many factors involved in hearing loss among these professionals, such as the engine in the front of the vehicle, and the loud noise in urban environments5. Many studies have reported NIHL among bus drives, with reported prevalences of 32.6% to 55.4%^6^, ^7^, ^8^, ^9^, ^10^. In the study carried out by Fernandes et al.^9^, in which all the buses had a front engine, hearing loss was worse in 49.1% of the right ears and in 62.8% of the left years.

Today, tonal audiometry is the most used complementary test to detect noise induced hearing loss. When necessary, one must use other complementary tests, such as immittance, logoaudiometry, evoked otoacoustic emissions (EOE) and the Brainstem Evoked Auditory Potential (BEAP) ^4^, ^11^.

The BEAP is a simple, objective and non-invasive text of the neural pathway which allows one to predict the psychoacoustic threshold through the electrophysiological threshold. In rare cases of simulation, it is not possible to trust the audiometric evaluation, since it depends on the patient's response. These situations require the otolaryngologist or the labor physician to perform objective tests, such as the BEAP and/or EOE. Therefore, it is fundamental to understand all the types of common electrophysiological responses found in NIHL individuals, and thus allow for a more accurate audiologic diagnosis.

Numerous studies have been carried out aiming at investigating BEAP alterations in patients with NIHL. Attias, Pratt^12^ did a prospective study of the changes in BEAP traces in 10 male machine operators aged between 18 and 21 years, exposed to noise of 112 to 117 dB (A) for eight hours daily, during 14 months. The authors observed that in the population studied there were modifications in the absolute latencies of the early waves; however, without significant influence on central neural conduction. Controlled clinical studies involving workers from many occupations, without prior history of ear disorders, under exposure to noise above 85 dB (A) for eight hours daily and exposure duration of more than five years, also did not find evidence of neural auditory pathway involvement among the patients in the study^13^, ^14^, ^15^. Noorhassim et al.^16^ evaluated the BEAP of 22 male workers between 50 and 69 years of age, from different occupations and at least 10 years of exposure to noise in their work environment to more than 90 dB (A). The BEAP was done and its responses were compared with the device's standardized data. Of the BEAP analyzed, 72.7% (32/44) were altered, and the prevalence of alterations was higher among those patients with the more severe hearing loss levels. There was an increase in the absolute latency mean values of LIP III-V and LIP I-V. the authors associated the BEAP electrophysiological alterations to the neural auditory pathway damage caused by noise exposure. Because of the age range studied (60 ± 8.7 years), the possibility of presbycusis as a contributing factor was not ruled out. Attias et al.^17^ assessed, in a clinical case/control study, the BEAP waves of 13 individuals (21 ears) between 21 and 45 years of age, without a past of ear disorder and with noise-induced hearing loss. The patients evaluated had different occupations and exposure to noises above 85 dB (A) for more than five years. In the study group we observed potentials with significantly lower mean amplitudes and significantly more prolonged PIII and LIP I-III absolute latency mean values. The authors then concluded that in the study group there was functional damage to the neural auditory pathway proximal to the inner ear. Thakur et al.^18^, in a clinical case/control study, assessed the BEAP of 24 male subjects with mean age of 48 years (± 7.593), who worked in different sectors of electrical and mechanical maintenance at the Mumbai airport, with mean sound exposure level of 95 to 110 dB (A) for eight hours daily during 15 to 30 years. In the study group they found a statistically significant extension of the mean absolute latencies of PIII in the right ear and of PIII, PV and LIP I-III in the left ear. The authors concluded that high sound exposure for many years in the airport contributed to the alterations found in the BEAP of these workers, and that these suggested neural conduction involvement of the auditory pathways all the way to the high brainstem.

The lack of studies on the BEAP alterations in bus drivers with NIHL and the different results of the previous workers justified this study.

The goal of the present investigation was to assess, by means of the brainstem evoked auditory potential, the involvement of the absolute latencies of potentials PI, PIII and PV and LIP I-III, LIP III-V and LIP I-V in a sample of urban bus drives with NIHL, compared to a group of normal-hearing individuals without noise exposure, with the aim to check whether or not there is any neural auditory pathway involvement in the study group.

## MATERIALS AND METHODS

We carried out a prospective and controlled clinical study. This study was approved by the Research Ethics Committee of this institution under research project 16/06. After the approval we collected the data from October 2006 through January of 2008. The selection of patients was sequential for the study group and happened as the workers were referred from their bus company for a periodic audiometric test. For the control group we selected volunteers. All the subjects signed an informed consent form in order to take part in the study.

The study group (NIHL group) was made up of 50 male individuals (100 ears), aged between 27 and 40 years (mean age: 37.5 years), from a population of drivers of front-engine buses and mean daily noise exposure of 85 to 93 dB (A) during a work shift of 8 hours. Noise exposure data from these patients was obtained from observing dosimeter reports carried out by the work safety and engineering staffs of the company. For the control group (C.G), 20 normal-hearing male subjects aged between 29 and 40 years (mean age: 34.2 years) without a past of noise exposure were selected.

In the NIHL Group we included bus drivers according to the following inclusion criteria: 5 or more years of employment working with the company; no history of prior ear disorders; audiometry showing a sensorineural hearing loss only in sound frequencies between 3 and 6 kHz, bilaterally; tonal audiometry thresholds between 3 and 6 kHz below 45dB HL; percentage index of speech recognition above 90%; type A Jerger dynamic tympanometry with immittance and contralateral stapedial reflex of 0.5 to 4kHz; 14 hour auditory rest before auditory and BEAP tests. Exclusion criteria from the NIHL Group were: prior neurological disorders; another work and/or leisure activity with noise exposure; prior exposure to organic solvents.

For the control group (C.G) we selected individuals with the following conditions: no prior history of ear disorders; no prior exposure to noise and/or sound trauma; tonal auditory thresholds of at most 25 dB HL for all audiogram frequencies (0.25 to 8 kHz); speech recognition percentage index above 90%; immittance values with type A Jerger dynamic tympanometry and the presence of contralateral stapedial reflex of 0.5 to 4kHz.

The patients were evaluated by means of an interview – following a set of guidelines according to the inclusion and exclusion criteria. All the patients were submitted to otoscopy. Tonal audiometry was carried out by the ascending-descending approach, by air conduction in the frequencies of 0.25, 0.5, 1, 2, 3, 4, 6 and 8 kHz. When detected by the air conduction test a hearing threshold equal to or greater than 25 dB, a bone conduction test was carried out in the frequencies of 0.5, 1, 2, 3 and 4 kHz. The percentage index of speech recognition was studied with a comfortable intensity level for the patient, established from the mean values of the 0.5, 1, 2 kHz sound frequencies. Audiometry was carried out with the Interacoustics® AC33 audiometer and immittance values with the Interacoustics® AT 235 immittance tester, calibrated according to the Brazilian Calibration Network and the ISO 8253-1 standard. The BEAP was studied with the Smart Intelligent Hearing System® device - Siemens, inside a sound treated booth, with the patients comfortably seated in a reclining chair. We first cleaned the skin with ether and placed disposable Meditrace® electrodes in the regions corresponding to the mastoid apophysis (M1 and M2) and on the upper forehead (Fpz), according to the International System of Brain Electrodes 10/20. The maximum accepted electrical impedance between the electrodes was below 5 kOhms. For acoustic stimulation we used ER3A Insert Earphones. Stimulation was in a single ear, ipsilateral, made up of unfiltered clicks of rarefact polarity and with inter-stimulus frequency of 19.3/s to an intensity of 80 dB HL, and total number of stimuli of, at least 1.000 clicks. The rejection index accepted for the test was of a maximum of 20%. We executed two series of stimulus in each ear in order to certify BEAP reproducibility. All the tests were started on the right ear. At the end of the tests, the traces were analyzed as to the occurrence of PI, PIII and PV and as to absolute latency values and of LIPI-III, LIPIII-V and LIPI-V on the right and left ears.

For all the absolute latency variables and LIPs we calculated the mean and standard deviation values. In order to analyze the statistical difference between the right and left ears in both groups we applied the Wilcoxon test. Following, the variables from the control and NIHL groups were analyzed comparatively, in the study of statistically significant differences. The analysis of the differences between the groups was evaluated by the Mann-Whitney test. We used a significance level of 5% (? equals; 0.05) in all the statistical tests employed.

## RESULTS

In the NIHL group, the ear laterality by the Wilcoxon test of the tonal thresholds of the audiometric frequencies of 3, 4 and 6 kHz, showed that they were significantly higher in the left ear (Table 1).

**Table 1:** Mean and standard deviation of the tonal thresholds in the 3, 4 and 6khz audiometric frequencies and the mean values of the three frequencies, according to ear laterality in the NIHL G.

FREQUENCYTONAL	THRESHOLD MEAN VALUES (sd)	
	Right ear (sd)	Left ear (sd)
3 kHz	12,00 (+ 6,78)	21,40 (+ 6,85)
4 kHz	32,20 (+ 4,42)	40,90 (+ 5,22)
6 kHz	20,20 (+ 3,64)	27,40 (+ 5,07)
Mean 3, 4 e 6 kHz	21,47 (+ 3,80)	29,90 (+ 4,54)

“p” value: < 0.0000 for all the variables.

sd equals; standard deviation

In the control group, PI, PIII and PV potentials were identified in all the ears.

**Graph 1:**
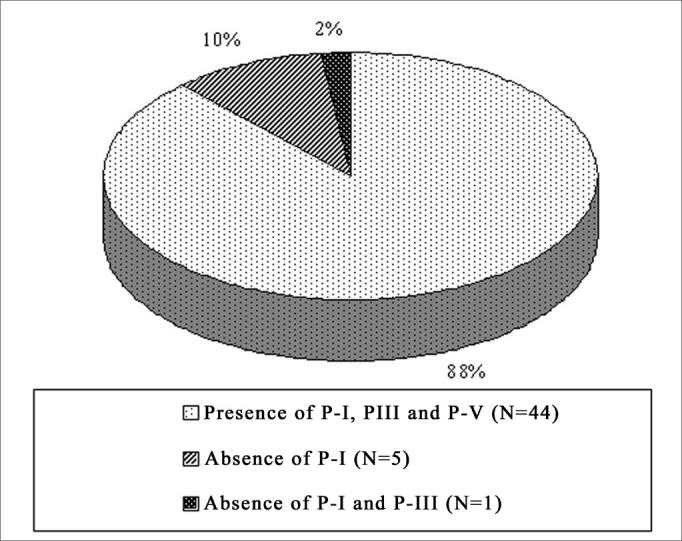
Right ear PI, PIII and PV prevalence in the NIHL Group

**Graph 2:**
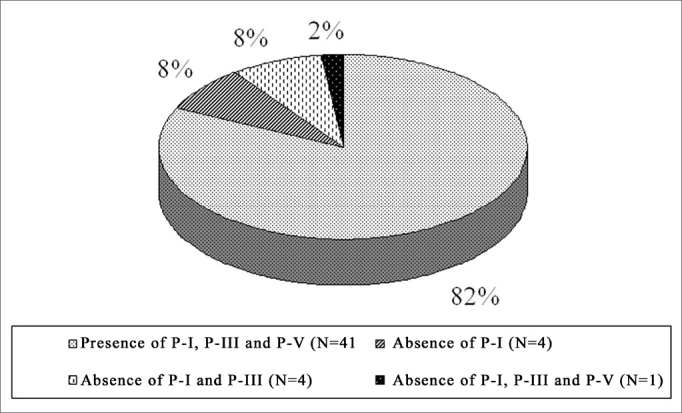
Left ear PI, PIII and PV prevalence in the NIHL Group

In the NIHL group, PI was not seen in 18% (n=9) of the ears, of which 10% (5/50) represented the right ear and 08% (4/50) the left. PI and PIII did not occur in 10% of the ears (n=5), of which 02% (1/50) to the right and 08% (04/50) to the left. PI, PIII and PV did not happen in 02% (01/50), on the left ears. These values are represented on Graph 1, Graph 2, separately, by ear laterality.

In order to analyze the statistical differences between right and left ear in each group, the Wilcoxon test showed that in both groups, C.G. (p equals; 0.0182) and NIHL G. (p equals; 0.0020), PI mean latency in the right ear was significantly higher than that on the left ear. In the NIHL G, the LIPI-III (p equals; 0. 0278) mean value on the left ear was significantly higher than that on the right ear. Table 2 shows the mean and standard deviation values of PI, PIII and PV absolute latencies and those for LIPI-III, LIPIII-V and LIPI-V, according to the ear and study group. Table 3 shows the “p” descriptive values, obtained with the Wilcoxon test.

**Table 2:** Mean and standard deviation of the absolute latencies of PI, PIII and PV and of LIPI-III, LIPIII-V and LIPI-V, according to ear laterality, in both groups.

Control Group					NIHL Group			
	Right ear		Left ear		Right ear		Left ear	
Latencies	N	Mean (sd)	N	Mean (sd)	N	Mean (sd)	N	Mean (sd)
PI	20	1,52 (+0,09)	20	1,47 (+0,08)	44	1,89 (+0,19)	41	1,81 (+0,18)
PIII	20	3,52 (+0,15)	20	3,55 (+0,22)	49	4,04 (+0,19)	45	4,02 (+0,18)
PV	20	5,52 (+0,18)	20	5,48 (+0,16)	50	5,96 (+0,25)	49	5,95 (+0,22)
LIP I-III	20	2,00 (+0,16)	20	2,08 (+0,17)	44	2,15 (+0,15)	41	2,21 (+0,18)
LIP III-V	20	1,99 (+0,28)	20	1,92 (+0,22)	49	1,92 (+0,24)	45	1,92 (+0,20)
LIP I-V	20	3,99 (+0,19)	20	4,01 (+0,16)	44	4,07 (+0,22)	41	4,13 (+0,21)

N equals; number of ears

sd equals; Standard deviation

**Table 3:** Descriptive levels of the Wilcoxon test (“p” values) obtained through the comparison of the absolute latencies and the LIPs according to laterality, in both groups.

Latencies	“p” values in both groups	
	Control Group	NIHL Group
PI	0,0182*	0,0020*
PIII	0,6631	0,1647
PV	0,1694	0,8705
LIP I-III	0,1985	0,0278*
LIP III-V	0,3505	0,6786
LIP I-V	0,9358	0,0525

“p” values with an asterisk: significant.

As to comparing mean latencies between the groups according to laterality, we observed a statistically significant extension of the three variables PI, PIII and PV in both ears of the NIHL group. (Table 4, Table 5)

**Table 4:** Distribution of the absolute latencies of PI, PIN and PV of the right ear, in both groups and the p value by the Mann-Whitney test.

	P_I_	P_III_	P_V_
Control G	1,52	3,52	5,52
NIHL G	1,89	4,05	6,00

“p” value: < 0.0000 for the three variables

**Table 5:** Distribution of the absolute latencies of PI, PIN and PV of the left ear, in both groups and “p” value by the Mann-Whitney test.

	P_I_	P_III_	P_V_
C.G.	1,47	3,45	5,48
NIHL G.	1,81	4,02	6,01

“p” value: < 0.0000 for the three variables

As to the comparison between the groups, once we consider the ear laterality and LIPs mean values, there was a LIPI-III extension in both ears and of LIPI-V on the left ear in the NIHL group. (Table 6, Table 7).

**Table 6:** Mean values of LIP I-III, LIP III-V and LIP I-V of the right ear in both groups.

	LIP I-III*	LIP III-V	LIP I-V
C.G.	2,00	1,99	3,99
NIHL G.	2,15	1,92	4,07

“p” value: 0.0010 (significant)

**Table 7:** Mean values of LIPI-III, LIPIII-V and LIPI-V of the left ear in both groups.

	LIP I-III*	LIP III-V	LIP I-V**
C.G.	2,08	1,92	4,01
NIHL G.	2,21	1,92	4,13

“p*”value: 0.0065 (significant)

“p***” value: 0.0128 (significant)

## DISCUSSION

Experimental studies showed neural structures involvement in NIHL^1^, ^2^, ^3^, probably secondary to hair cell degeneration and/or consequent to neural hyperactivity by the excess of acoustic stimulation. Many authors used BEAP to study whether or not there are evidences of neural involvement in NIHL individuals, but the results found were different because of the study methods and the sample profile^12^, ^18^. Moreover, in the papers by these authors, many occupations were considered as study goals; nonetheless, in the literature studied, the assessment of urban bus drivers was limited to the study of prevalence and NIHL risk factors^5^, ^10^.

The present study tried to assess a homogeneous sample, with the choice of subjects with the same occupation and, therefore, exposure to similar sound intensities; of similar audiometric profiles, characterized by mild sensorineural hearing loss, in order to avoid the difficulty in obtaining BEAP records in function of the NIHL of moderate or higher level; from a sample with age below 40 years in order to avoid influences from presbycusis eventually associated with NIHL. Immittance tests were carried out in order to check for tympanic-ossicular system functional integrity after 14 hour auditory rest, which was important to rule out transitional alterations of the auditory threshold. The work period choice above five years was based on the fact that most of the NIHL installs on the first five years of exposure^4^. In the present investigation, those individuals with previous neurological disorders and exposure to organic solvents were taken off the study in order to avoid the possibility of BEAP alterations stemming from these factors, concern which was also shown by numerous authors^13^,^14^,^17^,^18^.

The choice of audiometric profile of the patients in this study, characterized by sensorineural hearing loss restricted to 3 to 6 kHz frequencies and with tonal thresholds below 45 dB may represent a bias on the checked results, since it did not consider the evaluation of patients with NIHL in a moderate to severe level; Nonetheless, a greater reproducibility and sensitivity than BEAP on possible neural alterations in the first years of NIHL.

The mean values from the right ear PI latencies were significantly higher than those in the left ear in both groups. It is important to stress that, in the present investigation all the BEAPs were started systematically by the right ear, and such fact may have influenced the production of a potential with an extended latency in function of the most wakefulness state and or of greatest tension the patients suffer at the initial stage of trace recording.

In patients with NIHL, many authors observed a significant extension of the PI, PIII, PV and LIPI-III^12^,^17^,^18^ absolute latencies with results similar to that of the present paper. In this investigation, the NIHL group left ear presented LIPI-V mean value with a significant extension, at the expense of the LIPI-III extension. These data indicate that, besides the cochlear damage, there were damages to the neural synapses of the hair cells and the neural fibers of the cochlear nerve (1); the mean difference of LIPIII-V between the groups, control and NIHL, was not significant in both ears, which suggested anatomical and functional involvement of the neural pathways from the cochlear nucleus to the lateral meniscus (2); and that there was a greater functional involvement of the neuronal pathways to the left.

In the present investigation we identified the absence of PI in 18% of the ears, of PI and PIII in 10% and undetectable BEAP potentials in 2% of the NIHL G. This relatively low prevalence is justified by the fact that the sample was made out of patients with mild NIHL. The lack of PI and PIII was also noticed in different studies^13^, ^14^, ^15^, ^16^, and this lack was more frequent in more severe hearing losses. In these studies we noticed that neural conduction was not affected by the fact that the PV latencies were not extended, counterweighing the results found in the present investigation. In this, the lack of PI and or PIII potentials was more prevalent on the left ear, justified by its functional involvement.

Noorhassim et al. (1996)16 also observed PI, PIII and PV, and LIPI-V and LIPIII-V latency extensions, however, because of the age range studied (60 ± 8.7 years), the possibility of presbycusis has relevant. In the present study, in order to avoid such bias, we tried to assess subjects younger than forty years of age.

In this paper, the mean LIPI-V value was significantly more extended on the left ear, when compared to the control group, as established by Thakur et al.^18^. On the other hand, Xu et al. Observed mean values of LIPI-V within normal ranges in both ears^14^.

Many factors have contributed to the development of NIHL in urban bus drivers, such as the location of the engine in the front of the bus, the lack of proper sound insulation, the very age of the buses - usually above eight ears; the lack of preventive maintenance and the high level of urban noise – which the authors described as being higher than the acoustic comfort threshold^5^. As it happened in the present study, Fernandes et al.^9^ reported a higher NIHL prevalence in the left ears of urban bus drivers of front engine buses in the city of São Paulo.

In the city of Salvador, most urban buses do not have air conditioning, and such condition added to the climate characteristics of the place impact this fact because the vehicles run with the windows ajar, and such factor favored the propagation of noise to inside the vehicle. This fact must be considered as a possible cause of greater noise exposure to the left ear.

Thus, with this study we could infer that, besides the damage to the hair cells of the Organ of Corti, there was an early functional involvement of the neuronal auditory pathway of the lower brainstem, and these alterations were probably, proportional to the level of sound exposure.

## CONCLUSION

BEAP is a useful complementary test in the assessment of NIHL and shows early on that besides the sensorial damage, there is also damage to the first afferent neural pathways of the auditory system.
